# Dr. Parry's Elements of Pathology and Therapeutics

**Published:** 1816-03

**Authors:** 


### PART II.

### COMPREHENSIVE ANALYTICAL REVIEW OF MEDICAL LITERATURE.

" Tros, tyriusve, *nobis* nullo discrimine agetur."

### *Dr. Parry's Elements of Pathology and Therapeutics.*

( Continued from page 159. )

We shall pass entirely over two sections in our author's work—one on the structure and functions of the Nervous System; the other on the Mental Faculties. In the *first* are many accurate and ingenious anatomical remarks, tho' we think he has eulogised rather too highly, Messrs. Gall and Spurzheim. In the second, there is much acute reasoning, and much good sense. The following paragraph we extract for Mr. George Nesse Hill and his disciples.

" Although, however, sensations, and thence the ideas which grow out of them, are the materials from which we think, judge, and act, it is evident that they would little avail us, were there not *superadded* that capacity of *employing* them, which constitutes the faculties, by the degree or nature of which man is chiefly distinguished from other animals. P. 278.

### The Nervous Constitution.

The comprehensive term " *nervous*" has been applied to almost all inordinate movements of the parts concerned in the functions both of the animal and organic life, and to nearly all morbid states of sensation. This constitution shews itself by an extraordinary degree of sensibility and irritability; in consequence of which, certain impressions that, in a well adjusted constitution, are either indifferent or pleasurable, produce pain, inordinate actions, or both. Those who, from the habit of self-indulgence, the vicious compliance of parents, the indolence and luxury of wealth, or sedentary occupations, are exempted from the irritations and pains of body and mind, which Providence has made essential to our well being in this probationary state, are the certain victims to this Protean malady! The same causes produce nearly the same effects on various animals under the subjection of man.

As simple sensations become less acute by frequent excitement, we may readily conceive, that the causes which predispose to the nervous temperament, act immediately on the brain itself, as the ultimate organ of sensibility. It is true, that many parts of the organic life, as the heart, alimentary canal, &c. become inordinately irritable in nervous constitutions, but even here, we find, that the influence of mental impressions, acting through the brain on these various parts, is greatly lessened by the mere repetition of the irritation; so that a boy who starts with terror at the report of a gun, will, after having been a few weeks in the naval service, himself fire a cannon without the smallest trepidation.

Although original conformation may predispose individuals to be more or less easily acted on by the various causes of excitement, yet it is questionable, whether this state of morbid excitability, from long exemption from the causes of excitement, would reach that extent of disease which we see in the nervous temperament without the co-operation of some other cause.

"I think," says our author, "that such a concurrent cause does actually exist; and that this cause is excessive impetus of blood, acting on the medullary substance of the brain, or some other part of the encephalon." P. 292.

It must also be kept in mind, that increased impetus of blood not only excites actual disorder, but disposes the brain to be more easily acted on by other causes of irritation than if that excessive impetus of blood did not exist. And as this excessive determination to the brain does not, for the most part, produce its morbid effects until it has continued for some time, it is reasonable, thinks our author, to suppose, that this impetus is itself capable of aggravating or causing that inherent state of the brain termed original predisposition to excessive excitability. 294.

"If this be true, we may, perhaps, profitably carry our investigation one step farther, and inquire, whether the whole of this predisposition may not, in all cases, be formed through the medium of the *sanguiferous system;* so that the exemption from impressions, &c. above stated, as a cause of such diseases, may itself produce its primary influence on that system, while the brain may suffer only secondarily; but in its turn, re-act on the sanguiferous system. Thus, for the sake of illustration, let us suppose, that, from indolence or other causes, the heart has acquired an excessive morbid irritability. In this case, any impression communicated to it from the brain, may excite in it inordinate action, which determining the blood with excessive violence to the brain, may cause it to re-act on various other parts, and thus produce the phænomena of nervous diseases." 295.

Dr. Parry, so long back as the year 1788, attempted to prove that nearly all the modifications of nervous disorders originate in excessive momentum of blood in the vessels of the brain.* He there shewed that excessive sensibility in regard to external impressions, head-ache, vertigo, spasmodic dyspnœa, hiccup, general convulsions, and delirium, might be, for a while, wholly removed, or greatly mitigated, by compression of the carotid arteries. He asserts, that subsequent experience has added irresistible force to the above conclusion. Our author illustrates his pathological views by numerous apposite facts and observations. Thus the pulsation of the carotids in nervous persons, and in the nervous states of those persons, is preternaturally strong. The head is usually much hotter, and the face redder than in a state of health. *Insomnium* is brought on by excessive bodily or mental exertion, by anxiety, late hours, hot rooms, spectacles that strongly arrest the attention, frequent succession of objects which dazzle the eyes, &c. It is usually accompanied with cold feet, præternatural action of the heart, and throbbing of the carotids.

"Under these circumstances, sleep has been, on numerous occasions, induced by lying on one side, and making with the thumb a firm compression on one carotid artery."

Those strange noises which nervous persons are accustomed to hear, are supposed by our author to be occasioned by "the rush of arterial blood through some part of the vascular system of the ear," as they are apt to be produced by whatever increases the action of the heart, as hot rooms,

---

* Memoirs of the Medical Society of London, vol. iii, p. 77.

late hours, long watching, strong drink, violent muscular exertion, excessive mental attention, &c. and are diminished by all those causes which have a contrary tendency. When the rushing sound is waving or alternate, which it often is, it is exactly synchronous with the systoles of the heart. Our author has always been able to remove it entirely, *pro tempore*, and always to alleviate it, by compressing the carotid of that side. Nervous head-aches, whether affecting the external or internal part of the head, are owing to corresponding conditions of the circulation in the *external* or *internal* carotid. That which occurs from dyspepsia, or disordered peristaltic motion of the intestinal canal, is usually of the *first* kind, often extending itself to the muscles of the neck, accompanied with flushing of the face, strong pulsation of the carotids and their external branches. It may be relieved by strong pressure on the common trunks, in consequence of which, the peristaltic movement of the alimentary canal is often increased, the heat of the head is diminished, and the feet, if previously cold, become warm. The SICK HEAD-ACHE exemplifies that which arises from excessive determination of blood to the branches of the internal carotid. It is generally attributed to derangement in the liver or alimentary canal; but our author conceives that the state of the stomach is the *effect* not the *cause* of the malady in the head, which it *never precedes*, just as sickness and vomiting are the consequences and not the cause of the affection of the head, produced by a blow on the cranium.

"Accordingly, the sick head-ache may be cured or relieved by spontaneous bleeding from the nose, or other similar remedies applied to the head; but is not alleviated by purging, and is always aggravated by the stimulants which relieve dyspepsia."

Vertigo and epilepsy are explained on the same principles. The latter, says Dr. Parry, "whatever may be its primary causes, usually depends immediately on excessive impetus of blood in the vessels of the brain." Thus the symptoms of epilepsy are more or less of a convulsive, or even strongly contracted state of various muscles—chiefly those of the eyes, face, tongue, neck, throat, upper extremities and respiration, accompanied with loss of sense, and followed by stupor. Those parts are chiefly supplied with nerves from the encephalon, whose functions are greatly disturbed. The suddenness of the attacks, and the perfect intervals which exist between them, imply the operation of a fluctuating cause, which we cannot conceive to be any other than one of those sudden changes in the balance of the circulation which we continually see occurring in the sanguiferous system. When it occurs at an advanced age, it chiefly attacks those who have long been constitutionally nervous, or who have lost the accustomed excessive sanguineous determinations of gout, hæmorrhages from the nose, hæmorrhoids, ulcers, eruptions, &c.: and in all these cases, the pulse in the carotid arteries is habitually stronger than natural. Lastly, it often terminates in, or is exchanged for, sanguineous or serous extravasation in the brain, and consequent hemiplegia or apoplexy. Epileptic attacks are more or less prevented by whatever habitually diminishes the excessive action of the heart, or lessens the flow of blood to the head.

"Thus it is often superseded by gout. In more than one instance, the paroxysm has been removed by the affusion of cold water; and in others, by compressing the carotid arteries."

Dr. Parry very justly observes, that, where epilepsy arises from exostoses or other local diseases in the cranium, even here, the disease still attacks only in paroxysms; hence, the *local* disorders act merely as causes of predisposition, requiring for their production of the fit the coincidence of those causes which manifestly operate by increasing the impetus of blood to the head. But it may be doubted, whether these local derangements of structure also are not attributable to the same vascular impetus.

Convulsions are next adduced by Dr. Parry, as resulting from the same cause; and in fact, as a modification of epilepsy. Chorea is also considered by our author to be frequently dependent on the same cause, as are hysteria and hypochondriasis. Of Dr. Parry's opinions relative to *insanity*, we have availed ourselves in the review of Mr. Hill's work, and therefore shall omit them here. At page 346, Dr. Parry gives an explanation of what he means by particular determinations. He admits, (and we fully coincide with him) the existence of excessive *local* momentum without excessive *general* momentum.

"Thus I have many times known the pulse in the *temporal* artery so weak that blood would not flow from it, however well it was punctured; and other instances, in which it was too weak to be felt; and yet, in all, the pulse in the carotid artery has been extremely strong, and there has been the most decisive evidence of preternatural *impulse in the brain.* If, therefore, such a difference of impulse can exist in two sets of vessels derived from the same trunk, and so near to each other, we may readily conceive the internal branches and the capillaries arising from this artery, to be on other occasions, sufficiently full to produce all the symptoms, *without any increase* of fullness in the *trunk* of the carotid."

Hydrocephalus is another disease brought forward by our author, as exhibiting proofs of increased impetus to the brain; and also apoplexy. He thus concludes:

"From the foregoing relation of facts, I think it clearly appears, first, that a large proportion of nervous affections originates in a disordered state of the circulation with regard to the brain; just as inflammation, hæmorrhage, dropsy, and the various other maladies, which I have specified, arise from similar states of the circulation in other parts; and secondly, that this state is either relative, or absolute excess of momentum, impetus, or determination of blood, in some portion of the arterial system, of the part affected." 358.

Our own observations have long led us to conclusions nearly similar.

Speaking of tic douloureux, Dr. P. observes—

"All the circumstances induce me to attribute this pain to increased vascularity or determination of blood (perhaps amounting to inflammation) to the neurilema, or vascular membranous envelope of those nerves."

He thinks that the operation of cutting the nerve (performed by Dr. Haighton) was rather the division of the arterial branch supplying the affected ramification of the trigeminus nerve, than the division of that ramification itself.

A very interesting section follows, on "one common origin of diseases;" from which we shall extract as much as our narrowing limits will allow. As it is acknowledged that one family is more liable than another to scrofula, another to gout, a third to eruptive complaints, a fourth to mania, &c. so in different individuals of the *same* family there is a resemblance of modification in the several affections, proving them to be only varieties of the same common stock. Thus, in regard to the *head*, he has known one person maniacal; a paternal cousin hæmorrhagic and epileptic, and almost all his children subject either to epilepsy, head-ache, epistaxis, or hydrocephalus. In another family, the mother was *epileptic;* a son laboured under excruciating *head-aches;* a daughter died of *hydrocephalus.* Of two sisters, one had *eruptions* on the face; the other, flushing *heat of the head,* with nervous affections. Of two other sisters, one died (adult) of *hydrocephalus;* the other had *head ache, hysteria,* and *erysipelas.* In another family, a female had *epistaxis;* her sister had *nervous symptoms;* and two brothers were *maniacal.*

The extension of these diseases in different forms, and therefore under different names, to different parts nearly at the same time, is very interestingly illustrated by Dr. Parry. Many diseases appear to extend, by being joint affections of different or even remote branches of the same arterial trunks. Thus Dr. P. saw a man who, with violent rheumatic inflammation in the right shoulder, had a pulse in that wrist considerably fuller than in the other; that arm and hand were also hotter, and disposed to sweat, while the other was quite the reverse. When determination of blood takes place to the *bowels* in diarrhœas, &c. the muscles of the *thighs and legs* are often affected with pains, cramps, &c. and the feet are burning hot.

"During a gouty diathesis, a brisk purgative will often produce acutely inflammatory gout in the knees or feet." 372.

The purgative produces, we suppose, increased determination in the mesenteric and hypogastric arteries, and ultimately in the arteries supplying the lower extremities. Determinations of blood to the uterus, on the same principle, produce pains in the loins, groins, and down the thighs. These examples may suffice. Several curious examples of the "relation of diseases by remote changes" are adduced, of which we shall select a few.

Examples are very common where the same patients shall have, at different periods, hæmorrhoids, head-ache, vertigo, erysipelas, gout. Others, where a constitutional tendency to these will end in epilepsy, hemiplegia, or apoplexy. In one patient the succession was, gout, mania, and at last fatal epilepsy. In several instances, fits of epilepsy superseded gout. In a gentleman, an intemperate liver, gout, to which he was long subject, ceased, after an abscess with great discharge from the neck. In a gentleman, the cessation of gout was followed by cough, difficulty of breathing, anasarca, defective urine. These being cured, he was seized with a loss of sense, unattended by convulsions. He recovered, and was affected with apthæ all over his mouth and throat. No sooner was he recovered from this, than the original disease, gout, returned, aud became regular. Another patient had atonic gout, with quick pulse; defective, high-coloured urine; legs and thighs enormously swelled; and such a difficulty of breathing, apparently from hydrothorax, that for forty nights he had not even attempted to go into a bed: yet in a few days, by appropriate remedies, he lost every symptom of disease. And now a spontaneous and acute fit of gout came on, which terminated in perfect health. A lady, habitually subject to diarrhœa, fell into a state of costiveness. Some months afterwards, she was suddenly seized with giddiness and head-ache, accompanied with fever, followed by almost apoplectic insensibility, great heat of the head and face, with other symptoms of erysipelas. This disappeared after three or four days, and she returned to her former state of costiveness. Five months afterwards, pain and giddiness in the head, with erysipelas on the left side of the face, again occurred. And now there came on, by degrees, hemiplegia of the left side, together with loss of speech.

### Conversion of Diseases.

This Section is so extremely interesting, that we shall endeavour to condense as much as possible of it for our readers. — 1. In a gentleman, the pain of a node on the shin alternated with vertigo and a sense of numbness in the head. 2. A head-ache, of some years' duration, subsided, and was followed by a cough, and incessant wasting hectic fever. After the man had been long confined to bed, and death was every day expected, the head-ache began slightly to return; and as it became established, the cough and fever disappeared. 3. During vertiginous and other distressing complaints of the head, carditis twice or thrice occurred and suspended them; they immediately returned as the carditis abated. 4. Slight paralysis of the hands alternated with spasmodic asthma. 5. Vertigo and hæmorrhoids very commonly alternate. 6. In a gentleman, vertigo and head-ache were constantly relieved by œdematous swellings in the legs and feet. 7. In a lady, mania, ending in suicide, alternated with œdema of the ankles. 8. Fits of spasmodic asthma alternate with gout. 9. A gentleman lost epilepsy on being attacked with gout, a paroxysm of which was immediately followed by a sudden attack of asthma, which proved fatal in twenty minutes!

10. "On the other hand, various diseases of the head, as head-ache, vertigo, depression of spirits, mania, epilepsy, and apoplexy, in many instances, immediately or soon succeed the recession of inflammatory gout from the extremities." 382.

Let this be a caution to Dr. Kinglake, who endeavours to brow-beat every proof of the fatal effects of his favourite remedy. Vide *Med. and Phys. Journ.* for November, p. 361.

11. Recession of gout in a clergyman was followed by slight hæmorrhage from the rectum; which ceasing, fatal epilepsy supervened. 12. Epilepsy was superseded by pneumonia. 13. Vomiting of blood and mania alternated.

14. Bronchocele disappeared during hepatic inflammation. 15. Long-continued cough, hectic fever, emaciation, and night-sweats, ceased spontaneously on the breaking out of an ulcer on the scapula. 16. Orthopnœa and cough, of many years standing, suffered a sudden and violent aggravation. Œdematous swellings of the lower extremities and scrotum, with scanty urine, removed the orthopnœa entirely. After some weeks, the urinary secretion returned to the natural quantity, and the œdema vanished. Mental alienation now gradually succeeded, and, in a few months, gave way in its turn to asthma, which continued during the remainder of the gentleman's life. 17. The alternation of cutaneous eruptions with dyspepsia is well known. 18. That of the same disorders, with asthma and other forms of dyspnœa, has, in Dr. P's experience, been full as frequent, and much more important.

19. "I have often," says Dr. P. "seen various thoracic affections, as pulmonary consumption, asthma, carditis, or hydrothorax, arise from the spontaneous or artificial cure of ulcers, perpetual blisters, or fistulæ." 386.

It has lately been the fashion to ridicule these things; but we would seriously recommend the inexperienced and unobservant practitioner to ponder on the foregoing deduction from long experience.

20. Gout and erysipelas alternate. 21. An instance, where long-continued symptoms of apparently pulmonary hectic were entirely removed by a frequent and copious nasal hæmorrhage, which disease itself ultimately proved fatal. 22. Cough and bloody expectoration ceased on the supervention of œdema in the lower extremities; but returned, in a fatal degree, when the œdema vanished. 23. Many instances where extensive œdema ceased from violent spontaneous hæmorrhage. 24. Chronic bronchitis, or asthma humidum, is frequently relieved by œdema in the lower extremities. 25. A gentleman spat blood copiously every day for twenty years, during which he abstained from animal food and strong drink. Attempting to return to animal food by slow degrees, he had, in one year, four attacks of inflammatory fever. These were succeeded by vehement palpitation of the heart, which frequently returned during several years. They ceased on the supervention of cough and copious expectoration of mucus. After some years, the cough and expectoration disappeared, and were succeeded by dyspepsia and the original palpitation. These gave way to remedies, but were immediately followed by hæmoptoe. From this period, during the remainder of his life, which was extended to 80, the three states, of mucous expectoration, hæmoptoe, and palpitation, alternated with each other; but no two of them ever existed together. 26. Habitual cough, dyspnœa, expectoration, and deafness, were nearly cured by hemiplegia, but returned as the hemiplegia was relieved. 27. Habitual cough and expectoration were suspended by rheumatic pain on one side of the head, and returned as the latter disappeared. 28. Two instances occurred where the cessation of pleurisy was followed by peritonitis. 29. In several cases, pleurisy was converted into fatal inflammation of the cerebral coverings. 30. A gentleman was, for many years, so harassed by difficulty of breathing, cough, and copious expectoration, that he could scarcely ever lie down in bed. On his being seized with a painful erythema on the scalp, followed by deep sloughs, and accompanied with fever, the pulmonary symptoms entirely ceased. As the sloughs grew well, mania supervened; but, after a short time, was cured by low diet and depletion. From this time he remained free from complaint. 31. The disappearance of scarlatina is well known to be followed occasionally by bloody urine, arthritis, œdema of the extremities, ascites. To these may be added convulsions and epilepsy, both of which Dr. P. has seen. 32. A lady, for several years, had itching and smarting of the anus, with slight serous or mucous discharge. These disappeared, and were succeeded by violent catarrhal affection extending to the Eustachian tube; and when worst, to the bronchia, producing great stricture without cough. These symptoms continued long, and on the return of the affection of the anus disappeared. 33. A gentleman, who had long laboured under vomiting, was no sooner cured of it, than he became anasarcous. A spontaneous purging coming on, the anasarca disappeared. 34. Hæmorrhoids and rheumatism alternated.

35. "A gentleman had the following succession of maladies: Gout, often alternating with enteritis, followed by apoplexy and hemiplegia. The latter complaints were relieved. Then occurred enteritis, and in its place, an almost total want of the secretion of urine, without fever. This last symptom was succeeded by gout, which gave place to fever, attended with erysipelatous inflammation of the stomach, and fatal sanguineous vomiting, during which the urine was restored to its natural colour and quality." 392.

36. A girl, aged eight, had long a mucous discharge from the vagina. This ceased, and the eyelids became inflamed.

The latter malady disappearing, the former returned. 37. A lady, who, for many years, had been afflicted with cough and difficulty of breathing, was immediately and permanently cured by a large hæmorrhage from the humeral artery. 38. An old man, who had lived freely, had a chronic inflammation in one leg, accompanied with œdema. Both were greatly relieved by the application of a tight bandage. In a few days he was seized, for the first time, with epilepsy. 39 A young chlorotic lady had extensive œdema of the lower extremities. This was removed by bandages, when she was immediately seized with a painful affection of the right side of the head, which was always much relieved by a flow of tears from the eye of *that side*.

40. " A girl, seventeen years old, had a chronic ulceration of the foot. No sooner was this cured, than she was seized with a disease and enlargement of the heart, which proved fatal!"

41. In a lady, colica pictonum, with palsy of the hands, not arising from lead, were cured by the Bath waters. Four years afterwards, she had sciatica for five months. Stimulating friction immediately relieved the pain, but, in a few hours afterwards, was followed by a return of the colic, succeeded by palsy of the hands as before. 42. A gentleman had habitual excessive perspiration, which was cured. Immediately he became affected with hydrothorax, anasarca, and ascites; all of which, however, were happily removed by digitalis.

43. " In two cases, which occurred between twenty and thirty years ago, immersion of a gouty foot in cold water, which produced instant relief of the pain, and a proportionate abatement of the inflammation, was, in a few hours, followed by hemiplegia." 396.

Let Dr. Kinglake explain away these facts by a quibbling jargon of unintelligible reasoning! We again refer to his paper in the November *Med. and Phys. Journal* for an apology for the harsh expressions here used: but we cannot suppress our indignation when we see human life sported with to support a theory. Dr. K. seems to think, that the generality of practitioners adopt his treatment in gout. We appeal to the knowledge of every individual of the faculty, whether one in fifty of his acquaintances ever dreams of following Dr. Kinglake's plans.

Dr. Parry sums up the effects of *increased* momentum of blood in the following manner, viz:

" First, excessive determination or momentum of blood to the skin, produces—sweating, scarlatina, measles, erythema, erysipelas, and all the forms of eruptive diseases. Secondly, *to mucous membranes;* coryza, catarrh, whooping cough, croup, sore-throat, peripneumonia notha, catarrhus senilis; bronchitis, asthma; apthæ, dyspepsia, diarrhœa; and various other disorders of the villous coat of the alimentary canal; strictures in the urethra, œsophagus, colon, and rectum; gleet; fluor albus; catarrhus vesicæ. Thirdly, *to serous membranes;* phlegmon; pleurisy; pericarditis; peritonitis of different parts, constituting enteritis, puerperal fever, &c.; inflammation of the tunica vaginalis testis.—*To synovial membranes;* producing arthritis; together with the effects of these several states, anasarca, hydrothorax, hydropericardium, ascites, hydrocele, effusions into joints, adhesions, anchylosis, &c. &c. Fourthly, *to various other membranes;* of the spinal marrow or nerves, paraplegia, sciatica, tic douloureux, &c. *To the epithelion*, deafness. Fifthly, *to glandular parts;* cynanche parotidœa, or mumps; swelling and other disorders of the thyroid gland, mammæ, testicles, prostate, and various other glandular parts; phthisis pulmonalis; atrophy. Sixthly, *to the head;* head-ache, vertigo, sleeplessness, common nervous affections, mania, delirium, convulsions, hysteria, epilepsy, catalepsy, inflammation of the pia mater, or arachnoides; together with their occasional sequelæ, hemiplegia, apoplexy, hydrocephalus, and other effusions. Seventhly, to other parts in various forms; peripneumony, enlargement of the heart, liver, spleen, kidnies, testicles, and uterus, with or without inflammation; fungus hæmatodes, ophthalmia, cataract, amaurosis, Eighthly, *various increased natural discharges*, not already specified; ptyalism, diabetes, lachrymatio. Ninthly, *morbid depositions*, not above arranged; scirrhosites, indurations, ossifications, chalk stone, biliary and renal calculi, and other hard deposits in different parts. Tenthly, *hæmorrhages*, from serous, mucous, or other membranes, or parenchyma; as from the nose, uvula, fauces, lungs, stomach, intestines, kidnies, bladder, uterus, vasa deferentia, skin, liver, &c. To which may be added the various forms of purpura."

Such are the chief examples, according to our author, of diseases in which increased impetus, momentum, or determination of blood, forms one essential link in the series of previous phænomena or causes. In what particular cases they may depend merely on excessive *local* momentum, and in what there is the coincidence of increased action of the heart, it may be difficult to decide. The same symptoms may arise under both states.

When the above list is compared with that of diseases from *deficient* momentum of blood, the contrast would surprize a Brunonian or routine practitioner, where every complaint exhibiting any trait of *debility* is attributed to want of blood—*poorness* of blood, &c.; instead of irregular determination and *partial* plethora.

We shall glance hastily at the principal diseases of *defec-*

*tive* determination. It may here be observed, however, that *excessive* determination to *one* part, must of necessity produce *diminished* determination to *others*; but the *former* is the most formidable, and eclipses the latter.

1st. As the blood is the material of growth and secretion, we may conclude, that where there is a *partial* defect of these, there is not a sufficient afflux of blood to the part. Thus muscles shrink when not used; the heart becomes flaccid and exhausted, from unknown causes or from obstruction in the coronary arteries. 2d. To the same may be attributed the sinking in of one eye; sudden greyness of the hair, &c. 3d. Dr. P. knew three instances of sudden and entire failure of the pulse in the humeral artery and its branches, while action of the heart and of the other arteries was perfect. 4th. Defective menstruation. 5th. It is highly probable that costiveness, arising from want of the due peristaltic motion of the alimentary canal, is dependent on the defect of a due momentum of blood in the arterial system of that part. Thus Dr. P. has often known compression of the carotid relieve headache and flushing of the face, increase the warmth of the lower extremities, and at the same time produce a glow in the stomach and bowels, and a sensible propulsion forwards of the contents of the intestinal canal. 6th. In mortification from the ligature of arteries. 7th. Syncope from hæmorrhage, or various other causes preventing an adequate quantity of blood flowing to the brain.* 8th. Those gradual hæmorrhages from the nose, stomach, and hæmorrhoidal or uterine vessels, where the patient dies—"with an accumulation of blood about the heart and lungs"—and probably he might have added the *brain*, as the experiments of Dr. Seeds would lead us to expect.

"These," says Dr. P. "are all the cases of disease, whether topical or general, arising from defective determination or supply of blood, that I think it necessary to particularize here." 407.

Far as we have exceeded our boundaries, we must yet spare a few pages for the last Section of this invaluable volume.

*Exemplifications of salutary processes.*—That power of the constitution, termed RE-ACTION, though derided by some of our sciolists in medicine, most obviously shews itself by an unusual redness and glow of warmth which are perceived in parts that have been preternaturally cooled; and evidently arises from an increased determination

* Dr. Seeds's experiments prove, that there is an accumulation of blood in the brain at these times.

of blood to the parts, succeeding a deficient afflux of that fluid. In youth and strong health, this reaction produces no inconvenience; but occasionally it proceeds a step farther than is necessary for the well being of the part, and some morbid affection follows. This morbid affection is inflammation, differing in name and symptoms according to the texture of the parts. There is no disease, in which the process of reaction is more apparent than in the common fit of an ague; in this process, one of the most conspicuous circumstances is the occurrence of *shivering* which, as a modification of exercise, our author ingeniously supposes to be an effort of Nature to restore the balance of the circulation and heat to parts in which both were defective.

Dr. Parry considers convulsions themselves as only another modification, and perhaps a greater degree of the same state of tremor, and that they are salutary efforts of Nature to restore the balance of the circulation, in epilepsy and hysteria for example.

"So, under various modifications of nervous diseases, the patient shall sometimes have vehement fits of spasmodic coughing, and at other times, frequent vomitings; one purpose of which several motions is, to drive forwards the blood in the veins, and thus to promote a free and equable circulation of that fluid throughout the system." Page 416.

This is a very different operation from that of heat, wine, full meals, certain passions, and various other stimuli. *These* indeed cause the heart to produce an inordinate momentum of blood in the several branches of the *arterial* system, and more especially of the head, while the *venous* system is only secondarily and imperfectly acted on. Exercise, on the contrary, by urging forwards the blood in the veins, permits a ready evacuation of arterial blood into those channels; just as opening a vein produces a quicker determination of blood from the neighbouring vessels.

"Under impressions of sorrow, suspence, &c. not only is the patient relieved by tears, which unload certain branches of the carotid artery, but considerable mental ease is obtained by that species of deep inspiration, called sighing, by which the right auricle, and therefore the jugular veins, and the whole nervous system of the brain, are, in an unusual degree, emptied of their blood." 418.

By what catenation such salutary efforts are excited in the system, our author pretends not to explain. The facts themselves are offered as suggestions for the purpose of inducing further enquiry.

An excellent exemplification of reaction is afforded in GOUT. Our author has already considered this malady as evacuating and depleting the system; here he views it in the light also of "a powerful means of restoring the due balance of the circulation, or at least of changing the direction of excessive momentum of blood." He has suggested, that while indolence and habitual indulgence dispose the body to fall into disease, from even a slight plethora, these two habits, growing so much out of the present construction of civilized society, are apt to produce plethora itself. The diseases thus produced, are evidently those of irregular determination of blood, especially to the *head* and *alimentary canal,* parts that almost invariably suffer previously to, or during the intervals of gouty paroxysms. This is evinced on one hand by flatulency, predominant acidity, heart-burn, irregularity of appetite and bowels, and different degrees of sickness: on the other, by listlessness, incapacity of attention, depression of spirits, dreaming sleep, weight or pain in the head, vertigo, &c. Now during this excessive determination of blood to these important organs of the animal frame, there is, often, an unusual degree of coldness in the lower extremities, naturally proceeding from the defective balance of circulation. "Such is the state of circulation in the extremities, which usually precedes, and probably causes, the *reaction* of the constitution," which reaction, in an early period of life, is sometimes a mere aching and preternatural heat of the extremities, and perhaps, occasional cramps; "but at more advanced periods, especially in persons who have been subject to excessive determinations of blood to the head and alimentary canal, producing the symptoms above described, the reaction goes on to the extent of causing gout, erysipelas, anasarca, or other inflammatory affections of the lower extremities." 424. In these paroxysms, the aid of the heart is usually, but not always contributed towards the restoration of the long *defective* determination; and by this process, as by that of ague, the constitution is, for a greater or less length of time, relieved from those disorders, or from that tendency to disorder, under which it had before suffered.

In the first beginning of gout, a very short period seems sufficient to restore the balance of the circulation, and the patient is absolved by one fit of inflammation of thirty-six or forty-eight hours duration in a single joint, which is followed by œdematous swellings, and the speedy recovery of health, But at a more advanced period of life, each attack of gout consists of several distinct inflammations of different parts, occurring in succession, with short intervals, during which, not only those parts of the extremities, which have *not* been affected, still remain preternaturally cold; but even the toes shall be cold, while the instep of the foot suffers burning heat. 427. Thus the disorder proceeds till, if the progress be favourable, a complete restoration of warmth in the extremities ensues. Thus while one final end of gout may be, the evacuation of the habit, and the consequent reduction of a plethora which is relatively excessive, another end is the restoration of the due balance of circulation previously determined in excess towards other and more vital parts. Such are the pathological views of our author on the important subject of gout; and as they are the deductions of experience in a mind of no ordinary power of discrimination, they are of more value than all the chaotic speculations on that disease with which the public have been nauseated, from the *water-works* at Taunton to the *quackeries* of Montpellier; all of which have the same lethian tendency, in the opinion of more than ourselves.

"If the representation, which has thus been given be just, we can well understand why many local diseases cannot be removed, or even in a certain degree checked, by local remedies, without the hazard of converting a topical into a more general malady, or of causing a constitutional effort on some other part; which part may be more essential to life, than that which the attempt was made to relieve. The same evils may attend the administration of certain internal remedies, the tendency of which is not to cure the constitution, and so remove the necessity of the local disease, but merely to check the present salutary action of the system, and thus to cause only a temporary and delusive supension of present suffering. Such, in the far greater number of instances, is precisely the action of the *Eau Medicinale* of Husson; the injurious, and even fatal effects of which, local circumstances give me peculiar opportunities of witnessing."

We have now closed the longest analysis that perhaps has ever been given of a Medical Work in any Review. The ideas and the facts of our author were in such a state of concentration, that it required unusual exertions on our part, to exhibit the prominent features of this excellent work in any reasonable compass. Whether our labours shall be discriminated from the common mode of stringing together a series of long and unconnected extracts by aukward interpolations, we leave to time and the public. To those who can afford to place the *original* in their libraries, we think we have offered the most powerful inducement; to those who *cannot*—the most efficient succedaneum. To the author we can only offer our thanks—the three first numbers of the Medico-Chirurgical Review contain ample proofs of our unlimited admiration and esteem. In common with the medical republic, we shall anxiously look for the succeeding volumes.

---

### *Commentaries on the Diseases of Children.*

By Dr. Clarke.

